# Wool-Sorters Disease—The Hog Plague—A New Anthelmintic—Contractility of Capil Ary Blood Vessels—Production of Sex at Will—Sheep Rot—Microscopic Organisms in Ice—Traumatism of the Muscles of the Sub-Lumbar Region—A Celosomian Monstrosity of the Calf—Scabies—Curare—Carnivorous Habits of Bees—The Germ Disease Theory—Growth as a Function of Cells—Regeneration of the Vetreous Humor in Animals—Placenta of E Ephas—A New Preservative Fluid—Quinine for Hypodermic Use—Gelatine Suppositories—Kidney Worm—Pamphlet Box

**Published:** 1880-07

**Authors:** 


### PROGRESS OF VETERINARY SCIENCE.

"WOOL-SORTER'S DISEASES."—Quite recently five cases of this disease have occurred in the vicinity of Bradford, England, four of which have proved fatal. All the cases were men employed in sorting what is known as "Van Mohair." Malignant pustule formed in three cases. The physician under whose care one of these cases fell, and which was *not* attended with pustule, found bacillus anthraces in the blood after death, and certified the case as one of splenic fever. Previous examinations of the blood in these cases have shown this disease to be anthrax.—*London Lancet.*

THE HOG PLAGUE.—[Prominent Symptom taken from Agricultural Report, 1878.]—The hog plague is a disease *sui generis*, peculiar to swine, and owes its infectious character to the presence of a microscopic germ, the *bacillus suis*, which is found in all the fluids of the diseased animal. A period of incubation is required for its development, lasting from three to seven days.

The diagnosis is very easy. As symptoms of special diagnostic value, scarcely ever absent in any case, may be mentioned the following: Drooping of the ears and head; cough; dull look of the eyes; staring coat of hair; want of appetite; vitiated appetite for excrements; rapid emaciation; great debility; weak, sometimes staggering gait; tendency to seclusion; disposition to hide the snout or the whole head in the bedding; offensive smell and peculiar odor of the excrements, no matter whether constipation or diarrhœa exists.

The disease is extremely fatal, the younger the herd the worse. Few hogs recover from it, and those that do are comparatively worthless for the purposes of fattening and the slaughter house.

The morbid changes disclosed by a *post mortem* examination show hepatization of of the lungs to a greater or less extent; blood, serum and exudation in the pulmonal tissue; lymphatic and mesenteric glands enlarged; trachea and bronchial tubes full of frothy mucus; heart flabby and dilated in some cases, but in most, congested: congestion also of the liver, spleen, and kidneys; large and small intestines filled with humors of every size and shape, in many cases ulcerated; extensive adhesions of the bowels, agglutinations; quantitative and qualitative changes in the blood and other fluids, *bacelle* and *bacellus germs* everywhere.

A NEW ANTHELMINTIC.—The *ocinum basilicum*, a plant known in Buenos Ayres under the name "albochaca," has an action of such a nature that worms in every stage of development rapidly leave their location after the juice reaches them. Its use is so much the more to be recommended, since in the event no worms are present, no injurious effects result from the plant, but a laxative and disinfectant action is the only result. Fifty grammes of the juice is given, followed in two hours by a dose of castor oil. A free discharge of the worms may be expected.

The above observations of Dr. Lemos and the results obtained are very encouraging, and invite further investigation, the more since the number of anthelmintics is limited, and their action often unsatisfactory.—*Med. Neuigk.*, No. 34, 1879.

ON THE CONTRACTILITY OF THE CAPILLARY BLOOD VESSELS.—Five years ago M. Rouget presented to the Paris Academy of Sciences his observations on capillary contractility. He had found that the capillary blood vessels in the tails of certain tadpoles possessed contractile powers. This property they owed to the presence of ramified cells disposed in networks, and constituting a part of their walls. Recent observations on the blood vessels of the capsulo-pupillary membrane of newly born or fœtal mammals have confirmed Dr. Rouget's previous statements. He was able to observe the same phenomena here, as in the tails of various batrachian larvæ.

The endothelial tube, composed of flat nucleated cells, which ordinarily constitutes the walls of capillaries, was surrounded by a network of ramifying cells, and this latter imparted to these vessels the contractile quality.

The author also calls attention to the fact, that without the power of contraction, the capillaries could not, after death, empty themselves of their contents.—*Annales de Medecine Veterinaire, Jan.*, 1880. E. C. W.

---

PRODUCTION OF SEX AT WILL.—Mr. Fiquet, a cattle-breeder of Texas, claims to have discovered a way by which either sex can be produced at will. His theory is, that when the female at time of coition is comparatively cold, and the male very passionate, a female will be the result; a reversal of the conditions will produce a male. Mr. Fiquet secures the proper conditions by feeding the bull, for example, on rich food previous to the coition; the cows at the same time being kept on low diet, and submitted to the gallantry of a castrated bull, until the excess of their passion has worked itself off. In all the experiments, numbering fifteen or twenty, so far tried he has been successful in predicting the sex.—*Boston Med. and Surg. Journal.*

---

DETERMINATIONS OF SEX IN UTERO.—It is generally admitted that all females in the animal kingdom enjoy sexual intercourse just subsequent to menstruation to a greater degree, and that the pleasurableness of the act is diminished in proportion to its removal from the period. Now, if this be true, it is not unlikely that the mother should transmit to her offspring her sexual peculiarities, and that, too, in proportion as her nervous forces are drawn upon in the sexual act; therefore the further the act is removed from the menstrual period the more males will be born, and as the impression on the mother's mind is diminished so are the chances in the production of a female lessened.

I am of the impression that in dairy districts the observance of the above law enables stock raisers to produce from eighty-five to ninety per cent. of a desired sex. In breeding the fox-hound, for which animal my love amounts almost to weakness, I have observed that the above law is the rule, and, to any one who is curious enough to investigate, he will find the following facts to be true, viz.: That if a male be born as an exception to the above rule or law, it will partake more of the peculiarities of its mother than that male which was conceived in obedience to the law. He is more likely to partake of the same color, his voice will be similar, and about eighty per cent. of this class of males will be provided with imperfectly developed mammæ, and that conical protuberance commonly called nipple. It will be found that the fema'e born as an exception to the rule will partake of the peculiarities of the sire.

There are two cases in nature in which the off-spring invariably partake of the peculiarities of the sire. I refer to the *mule* and *hinny;* but, believing as I do, I am convinced that they would be modified if particular attention were paid to the natural law.—*Medical Record*, June 12, 1880.

Sheep Rot.—For some time a great mortality has prevailed among sheep, and the destruction reported in something appalling. The malady is popularly known by a very old Saxon name, "rot," and is in reality due to the presence in the liver and hepatic canals of numbers of the Distoma hepaticum, a trematode entozoon, as well as the Distoma lanceolatum, also a member of the same order. These entozoa, from their resemblance to the fish called "flukes," have received the same name, and have particular predilection for the bilaiary apparatus, whose function they more or less destroy, and thus lead to the slow death of the sheep or other animals they may infest. After wet seasons, animals which have been pastured on tainted land are certain to suffer, from their having ingested with the herbage the ova of the Distoma. Pastures are tained by "fluke" infested sheep, which pass the mature worms or their ova with the fæces, and these lodge on or are washed into the ground. The worms, of course, die, and the ova within them are liberated; and these together with the free ova, appear to have not only a strong vital resistance to meteorological alternations, but also the good fortune to find a ready and acceptable intermediary host in the Limnæus minutes, a little mud-snail common everywhere, and particularly on wet land. This snail becomes possessed of a number of ova in its interior, and during damp weather it crawls from its breeding place in the ground up the stalks of grass and herbage, and is swallowed by the sheep or other herbivorous animal when they are grazing. Received at first into the stomach, the ova undergo partial development, and then find their way into the biliary canals. If their number is considerable, when they have attained their full growth they dilate and obstruct these canals, the walls of which become considerably thickened. During their development the secretion of bile becomes gradually diminished, and that fluid is viscid, like mucus, and altered in color; at the same time the parenchyma of the liver becomes atrophied from the compression the "flukes" exercise upon it, and it may even become disorganized. Hence results icterus, disturbance in nutrition, anæmia, dropsy, and a general cachectic condition.

Sheep are not the only victims which suffer from the Distoma, for during the present mortality, hares, rabbits, deer, and horses are said to have become infested, and died. The Distoma hepaticum has long been known to exist in the horse and ass, when they were allowed to pasture on unclean land during wet seasons.

Salt appears to be an excellent and well-know prophylactic agent, and even a curative one when the disease has not made much progress. This beneficial action of sodium chloride has been known almost from time immemorial, and the freedom from "rot" of sheep which have been pastured on salt marshes has been also recognized for centuries.

The flesh of sheep which have been affected with this verminous disease cannot be said to be positively dangerous as food, though it must be greatly reduced in nutritive properties, as well as in quality. The human being may receive and harbor the Distoma, a fact worthy of remembrance. The present mortality is likely to render sheep scarce and expensive in this country for some time, and still further darken the prospects of our agriculturists.—*London Lancet.*

MICROSCOPIC ORGANISMS IN ICE FORMED FROM STAGNANT WATER.—Owing to the unusually mild weather of the past winter, ice has been gathered quite extensively from stagnant water in canals and ponds.

Fragments taken from the interior of blocks which appear clean and transparent to the unassisted eye, when put under a magnifying power of 900 diameters, show bits of vegetable tissues and confervoid growths.

Water obtained from such ice, when allowed to become warm at the ordinary temperature of living rooms, deposited a sediment in which, after some hours, monads are easily discernable, with a power of 200 to 400 diameters. In such water, if allowed to stand a little longer, may be detected thrifty convervæ and animalculæ similar to those found in stagnant water.

These investigations indicate that freezing does not free water from filth due to the presence of sewerage or decaying vegetable matter, and that the germs from which animalculæ are developed, if not the animalculæ themselves in a quiescent state, are present in very much of the ice taken from stagnant water. This being the case, the use of such ice in drinking water is hazardous, to say the least.—*American Naturalist, May*, 1880.

---

TRAUMATISM OF THE MUSCLES OF THE SUB-LUMBAR REGION.—M. Dupont, of Bordeux (Arch. Vetér. d'Alfort), in an interesting article on this subject, attempts to throw some light on the confused state of our knowledge concerning rheumatism, paralysis, and muscular traumatisms of the sub-lumber region. The following is a *resume* of his conclusions:

1. Authors, observers and veterinary practitioners have hitherto frequently confounded traumatism of the sub-lumbar muscles with the various forms of rheumatism and palsy.

2. Such traumatism is commonly observed during severe winters, and generally affects horses used to draw heavy freight. It is caused by the repeated and rapid slipping or sliding backwards of the posterior limbs during work.

3. It may be distinguished from the affections with which it has been confounded by its sudden advent, the horse suddenly falling at the moment the rupture of the muscular fibres takes place.

4. The different varities of palsy and rheumatism that may sometimes occasion a similar break down of the horse, can be foreseen and may be avoided, by a proper attention to special symptoms manifested as the horses continue work. Such symptoms may occur one-half to one hour before the fall.

5. Autopsies have confirmed this distinction, and have elicited the following facts: In traumatism with rapid termination, the lesions were limited to tearing of muscular fibres in the psoas magnus, to local hemorrhages, peritoneal and pleural complications. In traumatism followed by slow death, we find also rupture of the muscle-fibres of the psoas magnus, more or less extensive engorgement of the other psoas, traces of hemorrhage, a separation of the elements of the blood, imbibition of the connective tissue, a red discoloration of the anterior femoral nerves, and such other alterations as result from a prolonged contact of extravasated blood with these parts. The lesions of the nerves are ordinarily limited to their intra-muscular portions. The nerve centres and nerve roots are always normal.

6. In paralysis the sub-lumbar region presents no muscular lesions similar to those found in traumatism of the proas magnus muscle. The nerve centres and the anterior and posterior nerves are alone affected. They are found in the various stages of softening, show a comingling of the gray and white matter, and atrophy of the spinal cord.

7. A large number of muscular traumatisms in this region, result in complete recovery, and in this respect resemble the cases of rheumatism. The palsies, on the other hand, are never cured. E. C. W.

---

A CELOSOMIAN MONSTROSITY OF THE CALF.—Wm. J. Arkcoll, V.S., reports in the *Veterinary Journal* of May, 1880, the monstrosity herewith described. He was called to a cow in labor, and found the following parts of the calf presenting the two fore-legs, two hind legs and the head. After succeeding in delivering the same, it presented the following appearance: The spine was curved backward so that the pelvis rested upon the shoulders. The abdominal walls seemed to be incomplete, having an opening into the abdomen from 12 to 14 inches in diameter. The intestines were found attached to the calf, but reaching to the uterine walls of the cow, to which they were attached also. The heart, lungs, liver, etc., were found floating in the uterine cavity.

---

SCABIES.—Scabies in animals, like scabies or itch in the human subject, owes its origin also to the presence of a microscopic insect, though not the same insect. Unlike the same disease in the human subject, it frequently proves fatal. Its prominent symptom is itching of the skin, denudation of the hair, caused in most instances by the rubbing of the animal against objects for relief; the skin also much thickened and corrugated, and on its surface an immense amount of furfuraceous scurf, which is more or less attached to the integument. The animal has sometimes severe purging, and sometimes the opposite condition of constipation. The most effective treatment seems to be thorough washing of the entire surface with soap and water—soft soap and soft water, slightly warmed. This is to be repeated; that is, the animal is to have two washings, at what interval is not stated. Afterward is to be applied a liniment, composed as follows: Ol. lini, ol. terebth., ol. picis., aa, with an addition of flor sulphur. This to remain for three days, and then washed off. Internally, mild saline aperients where constipation prevails, the tendency to this to be further obviated by feeding bran and carrots. Whether constipation or diarrhœa prevails, in either case give diffusible stimulants and tonics, including Liq. arsenicalis. The writer observed that it was transmissible from the equine to the human subject, but that it did not flourish, and that it died out in a short time. The symptoms on the human body were like that of Nettle rash, with intense and intolerable itching.—*Veterinary Journal, May, 1880.*

---

A good deal of new light has been lately thrown on the nature of the Indian poison, curare. The plants which yield it all belong to the genus *Strychnos*. M Planchon distinguishes four regions as centres of preparation of curare, for each of which a principal plant may be indicated. These are English Guiana, the region of the Upper Amazon, the region of the Rio Negro, and Upper French Guiana.—*American Naturalist, May, 1880.*

---

CARNIVOROUS HABITS OF BEES.—Apropos of the asserted killing and eating, by hive bees, of moths captured by the bladder-flower, *Physianthus*, I would like to call the attention of readers of the *Naturalist* to the following statement by Kirby and Spence in their Introduction to Entomology, Letter xx., p. 384 of the seventh edition: "Though the great mass of the food of bees is collected from flowers, they do not wholly confine themselves to a vegetable diet; for, besides the honeyed secretion of the Aphides, the possession of which they will sometimes dispute with the ants, upon particular occasions they will eat the eggs of the queen. They are very fond also of the fluid that oozes from the cells of the pupæ, and will suck eagerly all that is fluid in their abdomen after they are destroyed by their rivals."—*Wm. Trelease, American Naturalist.*

---

THE GERM DISEASE THEORY.—A contribution to this subject has been made by Koch, who finds that certain species of bacteria, the lowest forms of plant life, occur in certain forms of disease in certain species of animals, and that such animals innoculated with such bacteria suffer from these diseases. Koch's method has been to innoculate mice or rabbits with decomposing animal matter, to notice what symptoms, if any, were the result of the operation, and to examine the tissues of the infected animal for the particular form of microphyte contained in the injected fluid. By injecting putrid blood or infusion of meat, and thus artificially producing septicæmia in mice, the animals died in a few hours, but it was found that the bacteria originally injected were still confined to the cellular tissue under the skin, and that they had not propagated themselves. It was also found that healthy animals innoculated with the blood of the dead animal were not injured by it. Here, then, the disease was evidently due not to living plants, but to a soluble poison—septin or sepsin—existing with the bacteria in the putrid fluid. But other symptoms set in about one-third of the cases, and it was found that one-tenth of a drop of blood from any part of an infected animal was able to communicate the disease to another. Thus Koch carried the disease through seventeen successive animals, the second being infected from the first, and so on through the entire series. An examination of the blood of any of these mice revealed multitudes of minute bacillus-like bacteria, of definite size and form, and evidently the contagium of this particular form of traumatic septicæmia, a disease peculiar to house mice. Beside the characteristic bacteria, occasionally a micrococcus-form was observed, which multiplied with great rapidity, forming characteristic chains in the subcutaneous tissue; the septicæmia-bacillus at the same time living and increasing in the blood. When injected into a mouse's ear these microcccci produced a perfectly distinctive disease, *i. e.*, necrosis of the tissues of the ear, which were penetrated through and through, and completely destroyed by the rapid multiplication of the micrococcus plants. This and experiments on rabbits and other mice showed that infection was produced by infinitesimal as well as by large doses; the bacterium-forms for each disease seemed thoroughly characteristic, the plants differing in size, mode of occurrence, etc., the presence of these microphytes being an indispensable requisite in the development of these symptoms. On the other hand, Dr. T. R. Lewis claims that one of the chief arguments against the germ disease theory, is the fact that a septiferous fluid retains its virulence after being boiled, filtered, evaporated, or combined with acids in the form of salts, but it is argued that this is not opposed to the action of a specific poison produced by the microphytes by a process of fermentation in the decomposing fluid —*American Naturalist, May, 1880.*

---

A New Theory of Gout —We notice in the *Medical Record* of April 17th, an article on the treatment of Subacute and Chronic Gout, by Alex. Hadden, M.D., visiting physician to the Presbyterian Hospital This malady, as is well understood by all competent practitioners, is one, in the treatment of which, attention to diet of the patient is an important consideration; indeed an essential point; the neglect of which may render nugatory any well considered course of medication. The distinctive features of Dr. Hadden's system of medication for gouty patients are the use of animal food in large proportion, and of such vegetables only as contain in small degree, or not at all, saccharine and amylaceous principles.

There are some maladies which are the result of a civilized condition; such may hence be termed diseases of civilization. Gout is, essentially, a disease of civilization, a malady which is unknown to the savage. Some of the animals which man employs in the civilized state, are also subject to certain maladies pertaining to that condition. It is a well known fact that certain domestic animals and birds, also certain wild animals and birds in a state of domestication, are subject to chalky and calcareous deposits in the joints and arteries, similar in character to the deposits found in the same localities in the human subject when suffering from gout. This being the case, the same remedial measures which experience has shown to be of service in the treatment of this abnormal condition in man, are found also serviceable in the treatment of domestic animals afflicted in the same way. Why not?

As stated in the article referred to, it is only the herbiverous and graniverous animals and birds that are subject to such deposits, while the carnivora escape them entirely. An argument is drawn from this by the writer in favor of his method of feeding the human subject when suffering from gouty deposits, upon a diet largely made up of animal food. In the case of any domestic animal of the grain or grass-eating species thus afflicted, while it may not be advisable to give them animal food, for such is contrary to their nature, it might be of advantage to feed to them such grains, grasses and other vegetable products, as contained in least amount, the saccharine and amylaceous principles in preference to such as are largely composed of these.

---

Regeneration of the Vitreous Humor in Animals.—Several years ago a communication on the reproduction of the crystalline lens was addressed to the Paris Biological Society. The same reporter has lately presented a paper embodying the results of experiments on the above subject. This gentleman operated on twelve young rabbits (aged four months), and twelve guinea-pigs, only one month old. The following method of operation was resorted to: A long transverse incision was made through the cornea, then by squeezing, the lens and vitreous humor were made to escape through the wound. Some of the latter was, however, allowed to remain in the eye-ball. One month later, the animals being in good health and their eyes appearing to be in good condition, one was killed in order to determine the precise condition of its eyes. The results of the examination showed a clear, transparent cornea and considerable new formed vitreous humor. Two months after this another rabbit was killed, and the operated eye showed a similar condition, only more advanced regeneration. After three months a third was examined, and in this one the vitreous humor was present in greater abundance on the operated than on the normal side. The experimenter was also struck by what appeared to be an incipient reproduction of the crystalline lens. The following conclusions of the author's various experiments are then given: 1. The vitreous humor may be regenerated after removal from living animals, provided only, a small amount is left behind. 2. The vitreous humor, in process of regeneration, may re-produce a capsule, and this in its turn form a true crystalline lens.—(*Gaz. Med. de Paris, quoted in Annales de Medecine Veterinaire, Janvier, 1880.*) E. C. W.

---

Dr. Chapman on the Placenta of Elephas.—The birth of an elephant at full term (twenty months and twenty days, according to the records kept by the keepers at Dr. Chapman's request), in Cooper & Bailey's menagerie in this city, afforded a unique opportunity to study the mature placenta of these huge animals. The placenta proper was found to be *zonary*, and was believed to have encircled the fœtal elephant during gestation. The amnion and chorion formed two large oblong pouches, one within the other, and were fused together equatorially at their narrowest diameters, the point where the placental villi were developed. On either side of the placental zone of villi, the numerous cotyledons were developed. The placentation was found to be essentially non-deciduate, and diffuse in character, with a zonary form; this combination of characters renders Dr Chapman's observations of great interest and systematic importance. No naturalist of recent times has ever had so good an opportunity to study this structure; the specimen described by Professor Owen is supposed, from its size, to have been immature, whilst the interpretations, figures, and descriptions of the parts by the older authors are necessarily unsatisfactory, owing to their lack of comparative knowledge.—*American Naturalist, May*, 1880.

---

A New Preservative Fluid.—About six months ago the German papers brought to notice that the conservator of the University of Berlin, Mr. Wickersheimer, had invented a fluid for preserving animal as well as vegetable tissues, which was said to surpass anything that had ever been used for that purpose. Mr. Wickersheimer's laboratory was reported to be the gathering-place of all sorts of scientists, who were unanimous in commending the extrordinary beauty and elegance of the specimen which Mr. Wickersheimer showed them, a number of which had been preserved for a considerable space of time. The well known naturalist, Carus Sterne, reported in No. 22 of the "*Gartenlaube*" as follows:

"Mr Wickersheimer has two ways of operating with his fluid. He either injects it into the veins of the body which is to be preserved, or soaks the whole object or any part of it in the fluid. By these methods the bodies are preserved from decomposition, and after having been taken out of the fluid and dried, their natural colors as well as the elasticity of the tissue and flexibility of all the joints are secured.

"Reporter saw the body of a boy which had died several months before, lying free in the open air and having perfectly preserved the appearance of a sleeping child. The body was of a natural softness and had preserved the appearance of life to a surprising degree.

"Mr. Wickersheimer showed a number of skeletons in which (the ligaments being preserved in their natural condition and elasticity) all the complicated movements could be executed and studied, of course much better than by aid of connecting wires and artificial joints. Some of the specimens showed beautifully the combined movements of the chest, the larynx and other parts in breathing. Several skeletons of snakes which had been treated with the fluid a year ago, allowed to show the spiral and undulatory movements of any part of the skeleton.

"But," the reporter continues, "not only the ligaments but also the vessels and membranes of animals will show the same indestructible softness and elasticity The lungs thus prepared in connection with the wind-pipe may, even after years, be inflated by means of b llows. Such old lungs of several animals reporter saw swelling to ten times their size; the lobes became distinctly separate; the brown color gradually changed into red, and at length the whole body appeared as if taken from out of a fresh body.

"Also the digestive organs after having been cleaned, prepared and blown up, may be transformed into durable preparations which are undoubtedly far more instructive than any of those common imitations in papier-machè.

"Further, the fluid offers great advantages for the preservation of such delicate objects which have to remain in a liquid medium. There is no discoloring, no skrinking of the object as in alcohol (even when diluted). Sections of delicate tissues, morbid formations which have been removed by an operation, will appear after months as if in a fresh condition, and may thus be preserved for further study.

"Finally, all sorts of vegetable organisms, such as flowers, fruits, fungi, etc., will excellently preserve in this fluid and are sure to maintain their natural appearance for a long time. Reporter saw a colony of those delicate common fresh water algæ which had been in the fluid for a year and had so beautifully preserved their green color that they appeared to grow in the water in their natural condition."

Some time previous to this report, Mr. Wickersheimer had offered his invention to the Prussian government for a reasonable compensation. The government accepted the offer and appointed a committee of experts to examine the fluid and test its qualities and effects. The very satisfactory results of these examinations have been quite recently published by the State's-Secretary of the Department of Instruction in the official "*Staatsanzeiger*," together with the following formula for the preparation of t e fluid:

In 3000 grammes of boiling water dissolve

| | | |
|---|---|---|
| Alum | 100 | grammes. |
| Common salt | 25 | " |
| Saltpetre | 12 | " |
| Potash | 60 | " |
| Arsenious acid | 10 | " |

After cooling and filtering, add to every ten litres of the solution, four litres of glycerine and one litre of methyl. alcohol.

The "*Staatsanzeiger*" says:

"The method of application differs according to the nature of the objects that are to be preserved. Anatomical preparations, whole bodies, etc., that are to be preserved dry, are laid (according to their size) from six to twelve days into the fluid, then taken out and dried in the open air. The ligaments, the muscles, etc., will now remain soft and flexible, so that at any time the natural movements can be executed.

"Hollow organs. such as the lungs, bowels, etc., must be filled with the preserving fluid, then laid in a vessel containing the same fluid and afterwards dried after the fluid has been poured out and the objects have been distended with air.

"Smaller animals, such as crabs, beetles, lizards, frogs, etc., if the natural colors are to be preserved unchanged, are not to be dried, but put up in the fluid

"If human or animal bodies are to be preserved for a longer space of time before they are used for scientific purposes, it is sufficient to inject the fluid. Two litres, f. i., will suffice for a child two years; about five litres are required for an adult. By this treatment the muscles will appear (even after years, when sections are made) as if in a fresh condition. If thus injected human bodies are preserved in the open air, they will gradually lose their fresh appearance and the epidermis will assume a brownish shade. But even this can be avoided if the fluid is externally rubbed into the skin, and if the access of air is prevented as far as possible

"This latter treatment may be recommended for corpses that are to be exhibited to the public or are to be preserved for some time before they are buried, for the features will remain unchanged in their expression and color, and there will not any smell be perceptible.

"For the real embalming a method of combined injection and preservation in the fluid is to be applied. The bodies, after being injected, are kept in tight cases, being wrapped in clothes which have been saturated with the solution."—*W. Barbeck, American Naturalist.*

---

Quinine for Hypodermic Use.—R. quinine sulphate, 1 drachm; acid lactici ft, 1 drachm; aquam distil ad, 3 drachms. The above formulæ, it is claimed, is not followed by ulceration.—*Va. Medical Monthly*

---

Gelatine Suppositories.—Pour six ounces of water over three ounces of gelatine in a water bath and let it stand until the gelatine has absorbed the water, then dissolve by heat and add eight ounces of glycerine continuing the heat sometime in order that as much water may be expelled as possible When cool, it can be reliquified and medicated as wanted Previously grease the mould with an oiled camels-hair pencil. —*Drug and Chem.*

---

Kidney Worm.—Recent investigations indicate that the kidney worm (*Strongylus gigas*) which exists in the kidney of the horse, dog, and sometimes in man, lives in fishes, in its young state, and the observations of Dr. Bertolas almost furnish proof that the people contract the Swiss or Broad tapeworm, (Bothriocephalus) by feasting on improperly cooked trout. According to Professor Van Beneden, however, the latter parasite is at present known to occur, only in Russia, Poland, and Switzerland. All full-grown fishes sold in the shops as food, are liable to contain entroyoa.—*So. Med. Record.*

INOCULATION OF RABIES.—Mr. Raymond inoculated a number of rabbits with the saliva and blood of a hydrophobic patient on the day before death.

The inoculations of blood gave negative results; the inoculations of saliva, however, were followed by rabies in a relatively short space of time, at most a few days. The submaxillary glands of two rabid rabbits inoculated on two healthy rabbits, produced in a short time the symptoms of rabies.—*Phila. Med. Times, Feb. 28th.*

---

SOLUTION OF SALICYLIC ACID.—Particularly beneficial in acute rheumatism. Recipe.—Acidi salicylici, 1½ ounces, Sodii biboratis, 2½ ounces, Glycerine, 3 ounces. Heat until dissolved, and add glycerine, 10 ounces, aquæ qs. s. ft. 3 pints.

Dose half pint every two or three hours till symptoms yield.

---

THE DOMESTICATION OF CERTAIN RUMINANTS AND AQUATIC BIRDS.—From n interesting correspondence between J. D. Caton, of Ottawa, Ill., and A. E. Brown, Superintendent Zoological Society, Philadelphia, contained in the June number of the *American Naturalist* for 1880, we gather the following: Mr. Brown's experience with our mule deer (C. Macrotis) has been similar to that of Mr. Caton, inasmuch as most of the animals died. Two bucks and one doe, however, which were first procured by the Philadelphia Society four years ago, have done well, and are in excellent condition. The others were all subject to diarrhœa, which was for a time checked by the use of astringent food, as oak leaves and rag weed; but in every case the disease returned. On post mortem examination a similar condition of things was found in every case.

The diarrhœa, it was ascertained, resulted from cancer of the stomach (except in the youngest animal, in which there was peritonitis, but no localized centre of irritation); the general physical condition was poor, tubercles generally being found in the liver and spleen; in each case death was immediately owing to the presence of a fibrous clot in the heart, resulting from the general impoverished condition of the animal. All had fed well or rather voraciously up to the day of death. The females have never appeared to take much care of the young, and the latter have been weaned very early. The attempts, therefore, to domesticate the species, were thus far a complete failure. Mr. Brown's experience with other species of deer, including moose, caribou, wapiti, mazaine deer, wood brocket, pudu, fallow, axir, sambur deer and others, are also stated. All of five specimens of moose and eight of caribou have died at periods varying from three months to two years and five months in the moose, and not beyond nine months in the caribou, from hypertrophy of the heart; owing, in his opinion, in great measures to the impossibility of providing the proper kind and quality of arboreal food, and somewhat also to the climate and the limited range given them in a zoological garden The wapiti and common deer have done well. The South American deer seemed to be constitutionally weak. Several mazaine deer, fallow deer, and sambur deer were bred and raised. The prong horn all speedily died from diarrhœa or hypertrophy of the heart.

Mr. Caton writes, that all his experiments with ruminants, *fera naturæ*, whose natural habitation is confined to the United States, west of the Missouri river, have proved failures. His mountain sheep died, and the Virginia deer continued to reduce in numbers His elk (*C. Canadensis*) still do well and are prolific. Concerning his efforts to acclimatize ornithological specimens, he says, that the Canada geese (*Bermicla Canadensis*), are very easily domesticated.

The white fronted geese (*auser cœrulescens*) do not domesticate so readily, and have not reproduced though they were observed to couple last spring. The Hawaiian geese (*Bermicla Sandvicenis*) have proved hardy, and came through the winter in good order. E. C. W.

